# Association between Lymph Node Status and Expression Levels of Androgen Receptor, miR-185, miR-205, and miR-21 in Breast Cancer Subtypes

**DOI:** 10.1155/2020/3259393

**Published:** 2020-04-23

**Authors:** Tatiana S. Kalinina, Vladislav V. Kononchuk, Alisa K. Yakovleva, Efim Y. Alekseenok, Sergey V. Sidorov, Lyudmila F. Gulyaeva

**Affiliations:** ^1^Institute of Molecular Biology and Biophysics-Subdivision of Federal Research Center of Fundamental and Translational Medicine, Timakova Str. 2/12, 630117 Novosibirsk, Russia; ^2^Novosibirsk State University, Pirogova Str. 1, 630090 Novosibirsk, Russia; ^3^Novosibirsk Municipal Budgetary Healthcare Institution “Municipal Clinical Hospital #1”, Zalessky Str. 6, 630047 Novosibirsk, Russia

## Abstract

Breast cancer is the most commonly diagnosed cancer among women. Difficulties in treating breast cancer are associated with the occurrence of metastases at early stages of disease, leading to its further progression. Recent studies have shown that changes in androgen receptor (AR) and microRNAs' expressions are associated with mammary gland carcinogenesis, in particular, with the formation of metastases. Thus, to identify novel metastatic markers, we evaluated the expression levels of AR; miR-185 and miR-205, both of which have been confirmed to target AR; and miR-21, transcription of which is regulated by AR, in breast cancer samples (*n* = 89). Here, we show that the molecular subtypes of breast cancer differ in the expression profiles of AR and AR-associated microRNAs. In addition, the expression of AR and these microRNAs may depend on the expression of PR, ER, and HER2 receptors. Our results show that the possibility of using AR and microRNAs as markers depends on the tumor subtype: a decrease in AR expression may be the marker for the presence of lymph node metastases in patients with HER2-positive subtypes of breast cancer, and disturbance of miR-205, miR-185, and miR-21 expressions may be the marker in patients with a luminal B HER2-positive subtype. Cases with metastases in this type of breast cancer are characterized by a higher level of miR-205 and a lower level of miR-185 and miR-21 in tumor tissues compared to nonmetastatic cases. A decrease in the miR-185 level is also associated with lymph node metastasis in luminal B HER2-negative breast cancer. Thus, the expression levels of AR, miR-185, miR-205, and miR-21 can serve as markers to predict cancer spread to the lymph node in luminal B- and HER2-positive subtypes of breast cancer.

## 1. Introduction

Breast cancer (BC) is the most commonly diagnosed cancer among women. According to the International Agency for Research on Cancer (IARC) estimates, around 2 million cases of this cancer type were detected in 2018 worldwide [1].

The end of XX century marks major advancement in BC treatment. Back then, the treatment method started to be determined based on the molecular classification proposed in 2000. This classification divides BC into subtypes depending on expression levels of estrogen receptor (ER), progesterone receptor (PR), Her2/neu receptor, and Ki67 [2]. But despite progress in the therapy and diagnosis of the disease, there are still significant difficulties in BC treatment, due in large part to the fact that nearly 30% of patients diagnosed with early-stage breast cancer will develop metastatic disease [3]. Lymph nodes are generally the first location of metastasis. Thus, in order to successfully combat this disease, early detection of metastases is absolutely essential.

Numerous studies have demonstrated that microRNAs can play an important role in cancer development and progression. MicroRNAs are short noncoding RNAs (19-25 nucleotides in length) that regulate gene expression through interaction with the mRNA-target. MicroRNAs can perform both tumor-suppressive and oncogenic functions and influence various processes associated with tumor development [4–6]. Lately, many publications have been focusing on the role of the androgen receptor (AR) in BC development and progression [7–9]. From these studies, androgen receptor expression became a novel prognostic factor.

In this study, we investigated the relationship between changes in expression levels of AR, miR-185, miR-205, for which AR is confirmed as a target, miR-21 (its transcription is regulated by AR), and tumor subtype (and its main characteristics such as T stage, N stage, and expression level of ER, PR, and HER2).

## 2. Materials and Methods

### 2.1. Tissue Samples and Definition of Breast Tumor Subtypes

All samples (pairs of the BC tissue and untransformed adjoining tissue samples) were obtained during surgery at the Novosibirsk Municipal Budgetary Healthcare Institution “Municipal Clinical Hospital #1” in 2017 (*n* = 89). Tissue samples were placed in an RNAlater™ Stabilization Solution (Invitrogen™, USA) and kept at -20°C until experiments were performed. All experimental procedures were approved by the Bioethics Committee of the Institute of Molecular Biology and Biophysics. Clinicopathologic information was obtained by reviewing medical records and reports on results of immunohistochemical assays. The following variables were determined: T stage, N stage, and IHC scoring of ER, PR, HER2, and Ki-67 expression (Table 1). Breast cancer subtypes were categorized according to the St. Gallen Expert Consensus as follows [10]: luminal A (ER+ and/or PR+, HER2−, and Ki − 67 < 14%), luminal B HER2-negative (ER+ and/or PR+, HER2−, and Ki − 67 ≥ 14%) luminal B HER2-positive (ER+ and/or PR+, HER2+), HER2-positive (ER−, PR−, and HER2+), and triple-negative (ER−, PR−, and HER2−).

### 2.2. RNA Isolation, cDNA Synthesis, and Real-Time PCR

RNA was isolated using TRIzol™ Reagent (Invitrogen™) according to the manufacturer's protocol. Glycogen (Thermo Scientific™, USA) was used as an RNA coprecipitant. RNA integrity was checked by running agarose gel electrophoresis. RNA concentration and purity were assessed using an Agilent-8453 spectrophotometer (Agilent Technologies, USA) at wavelengths of 260 and 280 nm. Reverse transcription was performed by using the RT-M-MuLV-RH kit (BiolabMix, Russia) according to the manufacturer's protocol. RNA at concentration of 0.8 *μ*g was used for reverse transcription reaction. cDNA was used in real-time PCR to measure the level of AR mRNA by adding a BioMaster HS-qPCR SYBR Blue (2x) (BiolabMix) reaction mix, followed by applying the CFX96™ Detection System (Bio-Rad Laboratories, USA). *SYMPK* and *POLR2A* were used as reference genes. The following specific primers were used in this study: *AR* 5′-CCTGGCTTCCGCAACTTACAC-3′, 5′-GGACTTGTGCATGCGGTACTCA-3′; *SYMPK* 5′-GCTGGAGAAGAAAGAGGTG-3′, 5′-ACAGGTTGGTGGCTTTGATG-3′; and *POLR2A* 5′-GCATGGCAGAGGAGTTTCGGCT-3′, 5′-ATTTCCCCGGGATGCGCAATGG-3′.

The optimal concentration for each primer was 300 nM.

Each PCR reaction was performed by using 0.3 *μ*l cDNA, at final volume 20 *μ*l, under the following conditions [12]: initial denaturation for 5 min at 95°C, followed by 40 cycles of denaturation for 15 s at 95°C, annealing for 20 s at 62°C, elongation, and fluorescence data processing for 30 s at 72°C. Melting profiles were used to assess PCR specificity. In each experiment, one plate contained samples of analyzed cDNA with a primer for AR gene and the reference genes (3 replicates per sample). The relative gene expression level was assessed based on threshold cycle (Ct) values considering PCR efficacy (E) for both the analyzed and reference genes.

### 2.3. MicroRNA Isolation, MicroRNA Reverse Transcription, and Real-Time PCR to Determine MicroRNA Levels

To isolate miRNA, a 50 mg tissue sample was combined with 500 *μ*l of guanidine lysis buffer (4 M guanidine isothiocyanate, 25 mM sodium citrate, 0.3% sarcosyl, 0.1% 2-mercaptoethanol, and 25 mM CH3COONa) [12]. Then, the solution was mixed and incubated for 10 min at 65°C. Samples were centrifuged for 2 min at 10000 g. Glycogen solution was added to the obtained supernatant, and the supernatant was combined with an equal volume of isopropanol, mixed, incubated for 5 min at room temperature, and centrifuged for 10 min at 10000 g. Then, the supernatant was decanted, and the pellet was washed with 500 *μ*l of 70% EtOH and 300 *μ*l of acetone, dried, and dissolved in 200 *μ*l of mQ-H2O.

The relative expression level for miR-205, miR-185, and miR-21 was measured using real-time reverse transcription-PCR. A reverse transcription reaction was performed using stem-loop-primers [13] and an RT-M-MuLV-RH kit (BiolabMix, Russia) according to the manufacturer's protocol. Real-time PCR was performed using TaqMan probes and PCR kits together with BioMaster UDG HS-qPCR (2x) fluorescence probes (BiolabMix, Russia) according to the manufacturer's protocol. To detect PCR products, the CFX96™ Detection System (Bio-Rad Laboratories, USA) was applied. Small nuclear RNA U44 and U48 were used to normalize the data.

Primers used for reverse transcription reaction for microRNAs: miR-205 5′-GTC GTA TCC AGT GCA GGG TCC GAG GTA TTC GCA CTG GAT ACG ACC AGA CTC C-3′, miR-185 5′-GTC GTA TCC AGT GCA GGG TCC GAG GTA TTC GCA CTG GAT ACG ACT CAG GAA C-3′, miR-21 5′-GTC GTA TCC AGT GCA GGG TCC GAG GTA TTC GCA CTG GAT ACG ACT CAA CAT C-3′, U44 5′-GTC GTA TCC AGT GCA GGG TCC GAG GTA TTC GCA CTG GAT ACG ACA GTC AGT T-3′, and U48 5′-GTC GTA TCC AGT GCA GGG TCC GAG GTA TTC GCA CTG GAT ACG AGA CGG TСA G-3′.

The primer sequences are based on the mature microRNAs' sequences taken from the miRBase database. To the 3′-end of the primer for the reverse transcription reaction, a microRNA-specific sequence of 6-8 nucleotides was added. The forward primer is complementary to 14-16 nucleotides at the 3′-end of the reverse transcription product.

In each experiment, analyzed cDNA together with primers specific to the target and reference snRNA was placed in the same 96-well plate (in triplicate for each sample). The relative expression level was assessed based on threshold cycle (Ct) values considering PCR efficacy (E) for both the analyzed and reference genes. The following specific primers were used: miR-205 (forward) 5′-GCCGCTCCTTCATTCCACC-3′, (probe) 5′-(R6G)-TTCGCACTGGATACGACCAGACTCC-(BHQ1)-3′; miR-185 (forward) 5′-GCCGCTGGAGAGAAAGGCA-3′, (probe) 5′-(R6G)-TTCGCACTGGATACGACTCAGGAAC-(BHQ1)-3′; miR-21 (forward) 5′-GCCGCTAGCTTATCAGACT-3′, (probe) 5′-(R6G)-TTCGCACTGGATACGACTCAACATC-(BHQ1)-3′; U44 (forward) 5′-GCCGCTCTTAATTAGCTCT-3′, (probe) 5′-(R6G)-TTCGCACTGGATACGACAGTCAGTT-(BHQ1)-3′; and U48 (forward) 5′-CCCTGAGTGTGTCGCTGATG-3′, (probe) 5′-(R6G)-TTCGCACTGGATACGAGACGGTСAG-(BHQ1)-3′. A similar type of reverse primer targeting the stem-loop region in the synthesized cDNAs was as follows: 5′-AGTGCAGGGTCCGAGGTA-3′.

## 3. Statistical Analysis

Data are presented as median values. Groups were compared using the nonparametric Mann–Whitney *U* test. A *p* < 0.05 was seen as statistically significant.

## 4. Results

### 4.1. Association of Expression Levels of miR-205, miR-185, miR-21, and AR with Breast Cancer Subtype

First, we examined AR and microRNAs' expression according to breast cancer subtype. We selected miR-205, miR-21, and miR-185 for analysis, since it was previously reported that AR is a target for miR-205 and miR-185 [14, 15] while transcription of miR-21 is regulated by AR [16]. The relative levels of AR mRNA and the abovementioned microRNAs were determined in 89 pairs of tumor and healthy tissues by RT-PCR (Table 2). We observed a significant decrease in miR-205 and miR-185 levels in the luminal B HER2-positive and luminal B HER2-negative subtypes. The expression of miR-205 was also decreased 5-fold in triple-negative BC. MiR-21 expression increased in all tumor subtypes. The level of AR mRNA was reduced 8-fold in triple-negative subtype and 3-fold in HER2-positive subtype.

### 4.2. Expression of miR-205, miR-185, miR-21, and AR in relation to Clinicopathologic Features of the Tumors

Next, we evaluated the relationship between the expression of microRNAs or AR and clinicopathologic features of the tumors (Table 3). We discovered that the level of miR-205 was associated with metastasis to the lymph nodes in the luminal B HER2-positive BC type. The level of this microRNA was higher in BC tissues of patients with lymph node metastasis compared to BC tissues of patients without. The miR-185 level was decreased in tissues of patients with lymph node metastasis in luminal B HER2-negative and luminal B HER2-positive BC types. In luminal B HER2-positive breast cancer, the level of miR-21 was also lower in samples of patients with metastases. A reduced level of AR mRNA was associated with metastasis to the lymph nodes in HER2-positive BC subtypes, both in ER-negative, PR-negative and ER-positive, PR-positive. In addition, there was a tendency to decrease the level of AR mRNA in BC tissues of patients with higher Ki-67 in ER-negative, PR-negative subtypes.

We also assessed the relationship between levels of microRNAs and AR mRNA and expression of ER, PR, and HER2. We discovered that for all luminal BC subtypes, the miR-205 level is significantly higher with a high value of PR expression (6-8 score according to the IHC assay) than with a 0-5 score of the expression value. In contrast, the expression level of miR-185 was lower with a high value of PR expression in luminal B HER2-negative and luminal B HER2-positive subtype of BC. Also, we detected an association between the expression of ER and PR and level of AR mRNA in luminal B HER2-negative subtype: the level of AR mRNA increased in samples with higher ER and PR expressions. The expression levels of miR-185 and miR-21 were associated with the expression level of HER2. So, in luminal B subtype, the level of these microRNAs was increased when evaluation of HER2 expression has a 2-3 score. A similar trend was detected for ER-negative, PR-negative HER2-positive BC subtype, but the difference in expression was not reliably significant.

## 5. Discussion

Significant progress has been made in the diagnosis and treatment of human malignancies, but cancer still remains to be one of the leading causes of death worldwide. Breast cancer is accounting for about 23% of cancer diagnoses in women [17]. The situation is complicated by the fact that about a third of women have metastases in the early stages of BC, and it leads to further relapse and progression of the disease [18]. Early detection of BC metastases is important for monitoring BC progression and predicting disease outcome. Thus, identifying markers that could indicate the presence of metastases remains a priority in the fight against this cancer. MicroRNAs have shown great potential as a new class of biomarkers in cancer. However, most current studies of the microRNA role in breast cancer are conducted regardless of the tumor subtype.

The androgen receptor is an important therapeutic target in the treatment of prostate cancer, but recent studies have shown that it can also have a therapeutic and prognostic value in BC [19]. Usually, the expression of AR is associated with a favorable prognosis in ER-positive tumors, while the results of studies of the role of AR in ER-negative, PR-negative tumors are controversial. Various authors have reported a relationship between AR positivity in a triple-negative subtype of breast cancer and a high or low frequency of lymph node metastasis [9; 20], high or low proliferative activity [7; 9], tumor size, and low survival rate [20]. Thus, AR can clearly play both tumor-suppressing and oncogenic roles in BC.

It is known that AR is expressed in almost all cases of ER-positive tumors; however, in ER-negative tumors, AR expression is predominantly observed in tumors of molecular subtype HER2+ [21]. The results of our study are consistent with previously obtained data. We showed that AR is expressed at a high level in the luminal subtypes, but receptor expression decreases 8-fold in the triple-negative subtype and 3-fold in the HER2-positive subtype. At the same time, the level of AR-regulating microRNAs (miR-205 and miR-185) was reduced in the luminal B tumors. The level of miR-205 also decreased in the triple-negative subtype, which corresponds to previously obtained data [22].

However, the actual task is to search for markers associated with the clinicopathologic features of the tumor. In our study, we discovered that decreased AR expression indicates lymph node metastasis in HER2-positive tumor subtypes. There were no changes in AR expression in tumor tissues of patients with metastases compared with tumor tissues of patients without lymph node metastases in other subtypes of BC. Moreover, the miR-205 level was significantly higher in tissues of patients with N1-N3 stage than in tissues of patients with N0 stage in the luminal B HER-2 positive subtype. It indicates a potential role of this microRNA in mechanisms of suppression of AR expression in this tumor subtype.

MicroRNA-185 is a well-studied tumor suppressor in breast cancer; the decreased level of which has been shown to be associated with the clinical stage and metastasis to the lymph nodes [23]. In this study, a decrease in this microRNA level was also observed in patients with lymph node metastases, but only in the luminal B HER2-negative and luminal B HER2-positive BC subtypes. A reduced level of this microRNA was also associated with T2-T4 stages in the luminal B HER2-positive subtype.

MicroRNA-21 is an oncogenic microRNA with an AR binding site located in its promoter [16]. The expression of miR-21 is activated by AR in human prostate cancer cells but decreases under the influence of androgens in breast cancer cells [24]. In our study, the level of miR-21 increased in all subtypes of BC but was significantly lower in tissues of patients that had lymph node metastases compared to BC patients without lymph node metastases in the luminal B HER2-positive subtype. A similar trend was observed for other subtypes.

Also, we found that the AR expression depends on PR and ER expression levels in the luminal B HER2-negative subtype: the AR mRNA level was increased in BC tissues of patients with a high expression of these receptors compared with BC tissues of patients with a low expression of PR and ER. The level of miR-205 also depended on the level of PR expression. The expression of miR-205 was higher in tumor tissues of patients with a PR expression IHC score of 6-8 than in tumor tissues of patients with a PR expression IHC score of 0-5 in all luminal subtypes. In contrast, in the luminal B subtypes, the miR-185 expression level was lower in BC tissues of patients who had a high PR expression value. According to the TargetScan database, PR is the potential target for miR-185. Thus, a decrease in the miR-185 level may be one of the mechanisms leading to disturbance of the PR expression in luminal subtypes.

The expression levels of miR-185 and miR-21 were associated with HER2 receptor expression and significantly increased in tumor tissues of patients who had a higher HER2 expression in the luminal B subtype. A similar trend was observed in patients with ER-negative, PR-negative HER2-positive BC.

## 6. Conclusions

Thus, our study demonstrated that the molecular subtypes of breast cancer differ in the expression profiles of AR and its associated miR-205, miR-185, and miR-21. In addition, the expression of AR and these microRNAs may depend on the expression of other receptors important for breast cancer control—PR, ER, and HER2. The possibility of using AR and miRNAs as markers to detect the presence of lymph node metastases also depends on the tumor subtype—a decrease in the level of AR expression can serve as a marker in HER2-positive subtypes, and a higher level of miR-205 expression and a decrease in miR-185 and miR-21 expression levels (compared with cases without metastases) may be a marker of metastasis in luminal B HER2-positive breast cancer. A decrease in the miRNA-185 level is also associated with lymph node metastasis in luminal B HER2-negative breast cancer.

## Figures and Tables

**Table 1:** Baseline characteristics of BC patients.

Characteristics	Luminal A (*n* = 24)	Luminal B HER2-negative (*n* = 20)	Luminal B HER2-positive (*n* = 20)	HER2-positive (*n* = 13)	Triple-negative (*n* = 12)
Age (mean and range, yr)	61 (41-78)	52 (27-83)	55 (39-70)	52 (42-61)	52 (40-74)
T stage					
T1	9	9	7	5	7
T2	15	10	12	7	4
T3	—	—	—	—	—
T4	—	1	1	1	1
N stage					
N0	16	11	8	7	9
N1	6	6	9	2	1
N2	—	3	1	3	2
N3	2	—	2	1	—
ER score					
0-2	—	3	1	—	—
3-5	2	5	3	—	—
6-8	22	12	16	—	—
PR score					
0-2	1	2	4	—	—
3-5	9	9	6	—	—
6-8	14	9	10	—	—
HER2 score					
1	—	—	13	5	—
2-3	—	—	7	8	—

Values are presented as the number of patients unless otherwise stated. ER and PR were graded using the Allred [11] scoring method.

**Table 2:** The association between the levels of miR-205, miR-185, miR-21, and AR mRNA in tissue samples from patients and subtype of breast cancer.

Subtype	Number of patients	Relative level^∗^ of miRNA or mRNA and *p* value							
		miR-205	*p* value	miR-185	*p* value	miR-21	*p* value	AR	*p* value
Luminal A	24	0.25 (0.09-2.14)	0.070	0.55 (0.12-2.03)	0.136	5.14 (0.79-35.15)	<0.001	0.85 (0.35-2.35)	0.295
Luminal B (HER2-negative)	20	0.21 (0.06-2.91)	0.041	0.25 (0.06-1.54)	0.005	3.53 (0.87-37.83)	<0.001	0.91 (0.44-2.35)	0.438
Luminal B (HER2-positive)	20	0.25 (0.03-1.56)	0.003	0.28 (0.08-3.16)	0.035	13.98 (0.39-64.68)	0.002	0.88 (0.29-2.40)	0.381
HER2-positive	13	0.52 (0.11-1.39)	0.115	0.48 (0.11-1.60)	0.115	6.68 (0.72-20.50)	0.018	0.33 (0.04-0.74)	0.001
Triple-negative	12	0.19 (0.03-0.94)	0.001	0.41 (0.09-1.42)	0.104	5.02 (0.81-21.54)	0.027	0.12 (0.01-0.31)	<0.001

^∗^Median and range of relative change of microRNA or mRNA levels in the breast tumor versus paired normal (adjoining) tissue.

**Table 3:** Association of miR-205, miR-185, miR-21, and AR expression levels with clinicopathologic characteristics in subtypes of breast cancer.

Characteristics		*N*	Relative level^∗^ of miRNA or mRNA and *p* value							
			miR-205	*p* value	miR-185	*p* value	miR-21	*p* value	AR	*p* value
Luminal A										

T stage	T1	9	0.96	0.132	0.41	0.895	10.02	0.433	0.92	0.332
	T2	15	0.25		0.79		4.58		0.84	

N stage	N0	16	0.45	0.867	0.55	0.632	7.10	0.285	0.82	0.824
	N1-N3	8	0.22		0.68		1.98		1.16	

PR score	0-5	10	0.12	0.006	0.41	0.365	2.92	0.423	0.69	0.151
	6-8	14	0.91		0.99		7.51		0.86	

ER score	0-5	2	0.37	0.412	1.30	0.208	14.61	0.952	0.84	0.960
	6-8	22	0.34		0.32		5.14		0.86	

Ki-67 index (%)	<10	10	0.22	0.594	0.18	0.082	6.68	0.689	0.81	0.765
	≥10	14	0.34		0.99		3.60		0.85	

Luminal B (HER2-negative)										

T stage	T1	9	0.46	0.082	0.22	0.138	4.17	0.832	1.17	0.399
	T2-T4	11	0.17		0.68		7.43		0.78	

N stage	N0	11	0.13	0.100	0.91	0.045	7.14	0.307	0.94	0.862
	N1-N2	9	0.35		0.13		2.89		0.97	

PR score	0-5	11	0.17	0.022	0.90	0.033	7.28	0.637	0.74	0.010
	6-8	9	1.89		0.13		4.17		1.57	

ER score	0-5	8	0.13	0.587	0.47	0.459	9.01	0.551	0.61	0.008
	6-8	12	0.23		0.14		5.01		1.47	

Ki-67 index (%)	<20	10	0.14	0.958	0.19	0.452	7.14	0.916	1.41	0.810
	≥20	10	0.17		0.88		4.17		0.89	

Luminal B (HER2-positive)										

T stage	T1	7	0.32	0.725	1.58	0.040	20.33	0.248	0.88	0.895
	T2-T4	13	0.18		0.20		4.05		0.89	

N stage	N0	8	0.08	0.008	1.03	0.036	36.07	0.045	1.17	0.036
	N1-N3	12	0.35		0.22		5.18		0.59	

PR score	0-5	10	0.12	0.043	1.92	0.031	23.82	0.431	0.80	0.930
	6-8	10	0.45		0.23		12.05		0.88	

ER score	0-5	4	0.16	0.585	1.50	0.845	11.79	0.585	0.76	0.764
	6-8	16	0.22		0.34		13.38		0.78	

HER2 score	1	13	0.35	0.116	0.31	0.018	3.05	0.006	0.88	0.693
	2-3	7	0.15		2.50		46.55		0.88	

Ki-67 index (%)	<20	9	0.18	0.927	0.24	0.262	9.29	0.525	0.80	0.653
	≥20	11	0.27		0.61		17.81		0.86	

HER2-positive										

T stage	T1	5	0.15	0.156	0.73	0.777	3.85	0.366	0.33	0.915
	T2-T4	8	1.01		0.21		10.17		0.26	

N stage	N0	7	1.07	0.897	0.43	0.713	9.28	0.270	0.39	0.022
	N1-N3	6	0.39		0.20		5.22		0.10	

HER2 score	1	5	0.18	0.167	0.27	0.167	5.53	0.915	0.12	0.362
	2-3	8	1.17		1.09		9.28		0.34	

Ki-67 index (%)	<40	6	1.17	0.235	0.57	0.523	6.68	0.835	0.43	0.095
	≥40	7	0.52		0.29		7.20		0.08	

Triple-negative										

T stage	T1	7	0.05	0.136	0.41	0.749	12.79	0.391	0.13	0.056
	T2-T4	5	0.27		0.49		4.59		0.03	

N stage	N0	9	0.13	0.171	0.24	0.362	5.02	0.868	0.13	0.820
	N1-N3	3	0.49		0.95		4.30		0.13	

Ki-67 index (%)	<70	4	0.16	0.915	0.81	0.110	9.91	0.156	0.15	0.110
	≥70	8	0.17		0.20		2.62		0.05	

^∗^Median of relative change of miRNA or mRNA levels in the breast tumor versus paired normal (adjoining) tissue.

## Data Availability

The data used to support the findings of this study are available from the corresponding author upon request.
